# Biomimetics: From Bioinformatics to Rational Design of Dendrimers as Gene Carriers

**DOI:** 10.1371/journal.pone.0138392

**Published:** 2015-09-18

**Authors:** Valeria Márquez-Miranda, María Belén Camarada, Ingrid Araya-Durán, Ignacio Varas-Concha, Daniel Eduardo Almonacid, Fernando Danilo González-Nilo

**Affiliations:** 1 Universidad Andres Bello, Facultad de Biología, Center for Bioinformatics and Integrative Biology (CBIB), Santiago, Chile; 2 Fundación Fraunhofer Chile Research, Las Condes, Chile; 3 Universidad Bernardo O Higgins, Laboratorio de Bionanotecnología, Santiago, Chile; 4 Centro Interdisciplinario de Neurociencia de Valparaíso, Facultad de Ciencias, Universidad de Valparaíso, Valparaíso, Chile; University of Quebec at Trois-Rivieres, CANADA

## Abstract

Biomimetics, or the use of principles of Nature for developing new materials, is a paradigm that could help Nanomedicine tremendously. One of the current challenges in Nanomedicine is the rational design of new efficient and safer gene carriers. Poly(amidoamine) (PAMAM) dendrimers are a well-known class of nanoparticles, extensively used as non-viral nucleic acid carriers, due to their positively charged end-groups. Yet, there are still several aspects that can be improved for their successful application in *in vitro* and *in vivo* systems, including their affinity for nucleic acids as well as lowering their cytotoxicity. In the search of new functional groups that could be used as new dendrimer-reactive groups, we followed a biomimetic approach to determine the amino acids with highest prevalence in protein-DNA interactions. Then we introduced them individually as terminal groups of dendrimers, generating a new class of nanoparticles. Molecular dynamics studies of two systems: PAMAM-Arg and PAMAM-Lys were also performed in order to describe the formation of complexes with DNA. Results confirmed that the introduction of amino acids as terminal groups in a dendrimer increases their affinity for DNA and the interactions in the complexes were characterized at atomic level. We end up by briefly discussing additional modifications that can be made to PAMAM dendrimers to turned them into promising new gene carriers.

## Introduction

Biomimetics is the implementation of principles from Nature to the development of new materials or systems. Evolutionary pressure has driven the optimization of efficiency in natural systems; thus, it is valuable to use this knowledge as a source of inspiration to solve existing problems[1]. One of the areas that can benefit from biomimetics is nanomedicine, which provides platforms to understand, build and use structures with biomedical applications at nanometric scale.

One of the current challenges in nanomedicine is the design of efficient and safe carriers for therapeutical agents, especially those based on nucleic acids, such as plasmid DNA (pDNA), antisense oligonucleotides and small-interfering RNA (siRNA), which have been considered as therapeutic macromolecules for prevention and treatment of several diseases[2]. Dendrimers are a class of nanoparticle that have gained prominence as efficient non-viral nucleic-acid carriers and drug delivery systems, because they posses terminal positive charges and unique features such as well-defined size and shape, monodispersity and variable end-groups[3–5]. Furthermore, dendrimers have been suggested as synthetic macromolecular analogs of proteins[6], due to their protein-like globular structure. Hence, dendrimers appear as appropriate scaffolds to incorporate the principles of biomimetic formulas.

During the last years, several efforts have been devoted to design dendrimers that can, on one hand, associate strongly enough to nucleic acids so that the latter remain intact during binding and entry into the cell (transfection process), and on the other hand, avoid induction of cytotoxicity which is a known issue of positively charged nanoparticles[7]. Several approaches such as acetylation or PEGylation[8] have been developed with the aim of decreasing cytotoxicity by introducing neutral end-groups, but at the cost of low transfection rates. Another issue that must be improved is how to modulate dendrimer-DNA interaction in order to promote the dissociation of the complex inside the cells, allowing the release of the cargo.

In search of new functional groups that could be used as new dendrimer-reactive groups to improve the interaction with nucleic acids, the incorporation of charged amino acids, such as Arginine and Lysine in poly(amidoamine) dendrimers has been proposed by several articles[9–13]. In those studies, terminal groups of poly(amidoamine) (PAMAM) of generation four were fully-substituted with Arginine or Lysine. In the first study reported by Choi and coworkers[9], the authors were based in the so-called “protein transduction domains”, which allow viruses to penetrate cell membranes. These domains, rich in positively charged amino acids such as Arginine and Lysine, served as an inspiration to build a dendrimer with these amino acids as end-groups. PAMAM-Arginine presented improved transfection efficiency in comparison to PAMAM-Lysine, PAMAM-amino terminated and commercial transfection reagents[9]. Based on the latter evidence, several research groups have proposed nucleic acids delivery systems based on PAMAM dendrimers modified with amino acids. Later articles have also recognized that, introducing amino acids, i.e. Histidine and mixtures of Arginine and hydrophobic groups such as Phenylalanine, could give dendrimers different properties that promote a better performance during transfection events such as endosomal escape ability[14] and improved interaction with cell membranes[15]. However, none of them have studied systematically or provided detailed information about the characterization at atomic level of the impact of inclusion of amino acids as end-groups in the dendrimer-nucleic acids interaction.

In this work, following a biomimetic approach, knowledge about how protein and nucleic acids interact with high prevalence in Nature was applied to identify new possible functional groups to be introduced to dendrimers in order to improve their efficiency as nucleic acids carriers. In a previous work reported by Luscombe et al. in 2001[16], the authors determined statistically the structural basis of the specificity between DNA bases and amino acids, using a dataset of all structurally-characterized protein-DNA complexes available at the time (129). For all interaction types, they concluded that over two thirds of contacts are made with the sugar-phosphate backbone of the DNA. Our aim was to update the survey carried out by Luscombe and colleagues on a much larger dataset of protein-DNA complexes now available, so as to identify the current trend of predominant amino acid residues involved in these types of interaction. For attaining this, we performed a statistical analysis of protein-DNA complexes whose structural atomic data were deposited in the Nucleic-Acid Database (http://ndbserver.rutgers.edu/)[17]. A total of 2,013 structures with a resolution lower than 3 Å were initially considered. Then, to remove redundancy, several subsets of protein-DNA complexes were generated, each of which consisted of non-homologous sets of proteins. Finally, the mean number of interactions and standard deviations for all sets was analyzed and reported.

Using this bioinformatics strategy, we have studied at structural level the most frequent amino acids observed in the contact zones of protein-DNA complexes: Arginine and Lysine. This knowledge was then applied for molecular design of new and more efficient gene carriers, which consist of dendrimer-based nanoparticles with amino acids conjugated on their surface that can act as customizable gene carriers. In this way, based on the natural behavior of the interaction between proteins and DNA, these new nanoparticles could be able to mimic better the surface properties of DNA-binding proteins. Besides, we report molecular dynamics studies to understand the complexation between a sample DNA fragment and amino acids-conjugated dendrimers, with the aim of determining if the incorporation of these biological groups increases the affinity of dendrimers towards nucleic acids and if these interactions show specificity for a certain nitrogenous base.

The strategy described here will allow the design of new synthetic proteins conjugated with different amino acids, turning dendrimers into new-engineered nanoparticles that can act as efficient and non-toxic nucleic acid carriers.

## Materials and Methods

### Structural dataset

In order to identify those amino acids with high frequency of interaction with nitrogenous bases in protein-DNA complexes, several searches were performed at the *Nucleic Acid Database*, NDB[17]. This public and free access database stores tridimensional structures of protein-nucleic acid complexes, generally obtained by X-ray crystallography or nuclear magnetic resonance. The search was focused on complexes of proteins and double stranded DNA (dsDNA). Only complexes with a resolution better than 3 Å were considered, resulting in an initial set of 2,013 complexes.

### Redundancy removal and generation of subsets to study

Starting with the 2,013 protein-dsDNA complexes, we used HBPLUS v3.0.6[18] to identify amino acid residues that were interacting with nucleotides in DNA (see below for parameters used). Those amino acids were then mapped to the domains to which they belong in the protein structures using the SUPERFAMILY assignment of domains[19]. Proteins with no domains assigned or those with protein-DNA interacting residues in domains not assigned were removed from the study, reducing the dataset to 1,623 complexes. By doing this, we identified several superfamilies that were over-represented in the dataset, including five that were present in more than one hundred of the remaining protein-DNA complexes: DNA/RNA polymerases (475 complexes), Lesion bypass DNA polymerase (Y-family) little finger domain (195 complexes), Ribonuclease H-like (165 complexes), Nucleotidyltransferase (149 complexes), PsbU/PolX domain-like (122 complexes). In order to avoid biases in the analyses we sought to generate a subset of protein-DNA complexes where only one representative domain per superfamily was allowed. Yet, the size of the resulting subsets was very small (65 to 71 complexes) and one such subset would have been too small a sample to represent the diversity present in the starting dataset. Thus, we generated 500 such subsets, assuming that the number of sets needed to find the best solution (x) can be approximated to -ln(p)/f, where p is the probability corresponding to the confidence level of having found the best solution, and f is the fraction of all possible subsets that produce the best solution. By setting p = f = 0.01, x approximates to 461. Because not all domains in a protein are assigned to a superfamily domain, we performed an all-against-all blastp search among all proteins in each of the resulting subsets to ensure that no homologous domains were left in the same subset. By doing this we identified a total of 7 sets (out of 500) where some proteins may have a distant homology relationship, and where thus eliminated. This resulted in a final dataset composed of 493 subsets of non-homologous protein-dsDNA complexes, which sampled 1,435 different protein-DNA complexes.

### Calculation of interactions

Once all biological unit structures of each protein-dsDNA complex were downloaded from the NDB server, hydrogen bonds and van der Waals (vdW) interactions were analyzed by means of the computational package HBPLUS v3.0.6[18]. The maximum bond distance between H-Acceptor was set at 2.7 Å, while the maximum donor-acceptor distance was adjusted at 3.35 Å. All the output files for the interaction analyzes were filtered to count only those interactions between amino acids and each of the nitrogenous bases, deoxyribose and oligonucleotide phosphates. In this study, besides side chain groups, primary amine and primary carboxyl groups of the main-chain of each amino acid were also considered in the interaction with DNA. For this reason, hydrogen bonds formation between aliphatic residues and DNA are also present, but in very low proportion. Moreover, data were not normalized over the abundance of each amino acid in the Protein-DNA contact zone, in order to keep information of their natural abundance in the studied structures.

The results reported correspond to the average number of interactions obtained for the 493 subsets studied. Data processing was accomplished using in-house computational scripts.

### Molecular simulations

Two molecular models of PAMAM G4 functionalized with 64 Arginines and 64 Lysines as terminal groups were built using home-made scripts. According to previous articles, only terminal groups of PAMAM remain protonated at neutral pH[20–22], so both dendrimers carry the same number of charges due to the terminal amino acids. Dendrimer models were later parameterized and adjusted to the CHARMM General force field (CGenff)[23], using the PARAMCHEM platform[24].

The 38 base-pairs DNA sequence used in this study, 5'-GCC-GCG-AGG-TGT-CAG-GGA-TTG-CAG-CCA-GCA-TCT-CGT-CG 3', has been reported and used as a model system in previous articles[25]. The CHARMM-force field, which has been refined for several biological molecules such as nucleotides and proteins, was set for the DNA model.

After dendrimer functionalization, a total of five molecular systems in TIP3P-water[26] boxes were generated: two systems considering each of the dendrimers, one system only with the DNA strand, and two systems containing each of the dendrimer-DNA complexes (Table 1). A concentration of 150 mM of NaCl was considered to simulate a physiological condition.

**Table 1:** Details of the molecular systems reported in this article.

System	Dendrimer charges	DNA charges	N° of Cl^-^ and Na^+^ ions	Total number of atoms	N° of water molecules
**G4-Arg + DNA**	128	-74	-263/+209	228889	74056
**G4-Lys + DNA**	128	-74	-188/+134	149312	47623
**G4-Lys**	128	-	-208/+80	88421	28139
**G4-Arg**	128	-	-231/+103	113006	36276
**DNA**	-	-74	-141/+215	152773	50004

All simulations were performed using the NAMD computational package[27]. Each system was first minimized through 2,000 steps until reaching convergence, and after that, molecular simulation runs were carried out for 50 ns at 310 K (dendrimer-only systems), 100 ns (DNA-strand system) and 180 ns (dendrimer-DNA systems). All simulations considered an NPT ensemble and periodic boundary conditions. Langevin dynamics with a damping coefficient of 1 ps and the Nose–Hoover Langevin piston[28] were applied for keeping the temperature and pressure (1 atm) constant. All hydrogen bonds were constrained during the MD simulations using the SHAKE algorithm. Long-range electrostatic interactions were calculated with the Particle mesh Ewald (PME)[29] algorithm and van der Waals forces were estimated using a cut-off of 10 Å. Equations of motion were integrated with a time step of 2 fs.

The MM-PB/GBSA (MM: Molecular Mechanics, PB: Poisson Boltzmann, GB: Generalized Born, SA: Surface Area) method[30, 31] has been extensively used to the study of energetics driving DNA-dendrimer complexation. Thus, the MM-GBSA method was employed, as our group has described elsewhere[4], to calculate binding free energy as the energy difference between the bound and unbound states of both molecules:

$$\Delta G_{bind}=G_{TOTAL}\left(complex\right)-G_{TOTAL}\left(dendrimer\right)-\;G_{TOTAL}\left(DNA\right)$$ (1)

According to the three-trajectories approach[32], which is appropriate due to the large amount of conformational states of the dendrimer, the free energy of each macromolecule: dendrimer, DNA strand and complex dendrimer–DNA was estimated as follows:

$$G_{TOTAL}=H_{MM}+G_{solv-p}+G_{solv-np}-T\Delta S_{conf}$$ (2)

The H_MM_ contribution corresponds to the sum of the terms calculated from the molecular dynamics trajectories, (E_bond_, E_angle_, E_torsion_, E_vdW_ and E_elec_), obtained from the last 10 ns of each MD trajectory of the dendrimer, DNA and complex dendrimer-DNA systems. Polar contributions to the solvation free energy (G_solv-p_) were solved using the Generalized Born approach, using the GBIS module included in NAMD, as a post-processing analysis of the MD trajectories. Non-polar contribution (G_solv-np_) was calculated as a linear function of the solvent-accessible surface area (SASA), considering a probe radius of 1.4 Å, using the following equation:

$$G_{solv-np}=\;\gamma SASA+\;\beta$$ (3)

where the parameters γ and β are standard values of 0.00542 kcal·mol^-1^ Å^-2^ and 0.92 kcal·mol^-1^ respectively[33], with SASA being obtained using the VMD software[34]. Finally, the entropic term was calculated employing quasi-harmonic analysis[35–38] implemented at the ptraj module[39] included in the AMBER[40] package. The ptraj module is suitable for the analysis of trajectories of several force-fields, including CHARMM-based NAMD trajectories. The quasi-harmonic analysis, using the Schlitter formula[41], considers configurational and vibrational modes arising from the complex formation, using a covariance matrix. The analysis was applied using 10 snapshots obtained from the last 10 ns of the MD trajectories.

## Results and Discussion

### Protein-dsDNA complexes analysis

Data of hydrogen bonds and vdW interactions in the protein-DNA complexes between each amino acid and phosphate groups (P), deoxyriboses (S) and each type of nitrogenous base: adenine (A), cytosine (C), guanine (G) and thymine (T) are described in detail in Table A in S1 File. Analysis of these results indicates that approximately 65% of the total number of hydrogen bonds established between amino acid residues and DNA occurs with phosphate groups, 23% with nitrogenous bases, and about 12% with deoxyribose. This evidence denotes that in protein-dsDNA complexes, the interaction between both types of macromolecules is dominated mainly by contacts with the deoxyribose-phosphate backbone, and therefore, the type of nitrogenous base has minor incidence in the generation of contacts between dsDNA and the protein.

**Fig 1:**
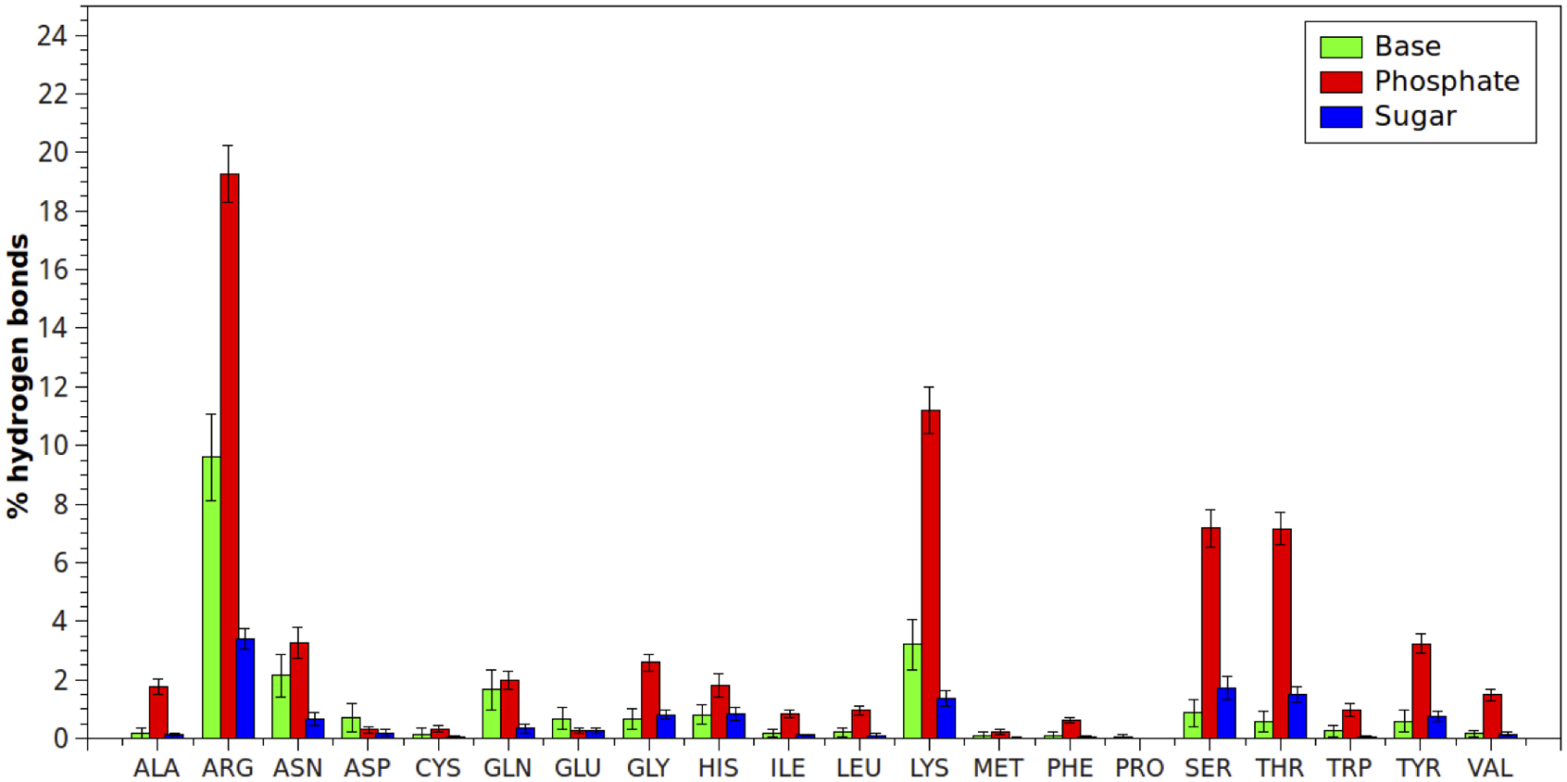
Hydrogen-bond interactions (expressed in percentage) established between each amino acid type and bases, sugar and phosphate DNA groups. Percentages were normalized considering the total number of hydrogen-bond interactions found in protein-dsDNA complexes.

**Fig 2:**
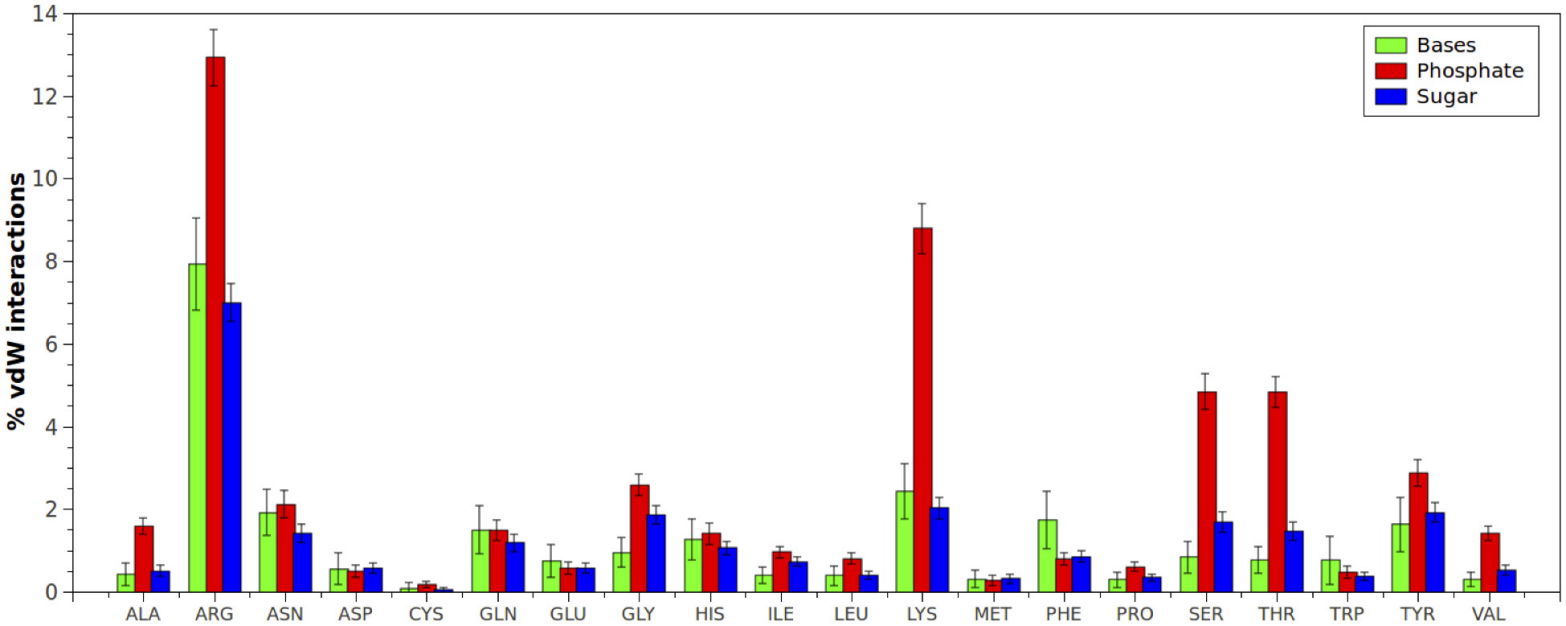
van der Waals interactions (expressed in percentages) established between each amino acid type and bases, sugar and phosphate DNA groups. Percentages were normalized considering the total number of van der Waals interactions found in protein-dsDNA complexes.

Percentages of hydrogen-bonds and vdW interactions between each amino acid type and bases, sugar and phosphate DNA groups were plotted in Figs 1 and 2. As can be seen, the most considerable interactions generated by the phosphate groups, both hydrogen-bonds and vdWs, were with Arginine and Lysine, followed by Serine and Threonine. The former amino acids have basic side chains at physiological pH, and therefore, the positively charged sites facilitate the initial electrostatic interaction with the phosphate group. The latter amino acids have neutral alcohol side chains, supporting both van der Waals and hydrogen-bonds interactions.

**Fig 3:**
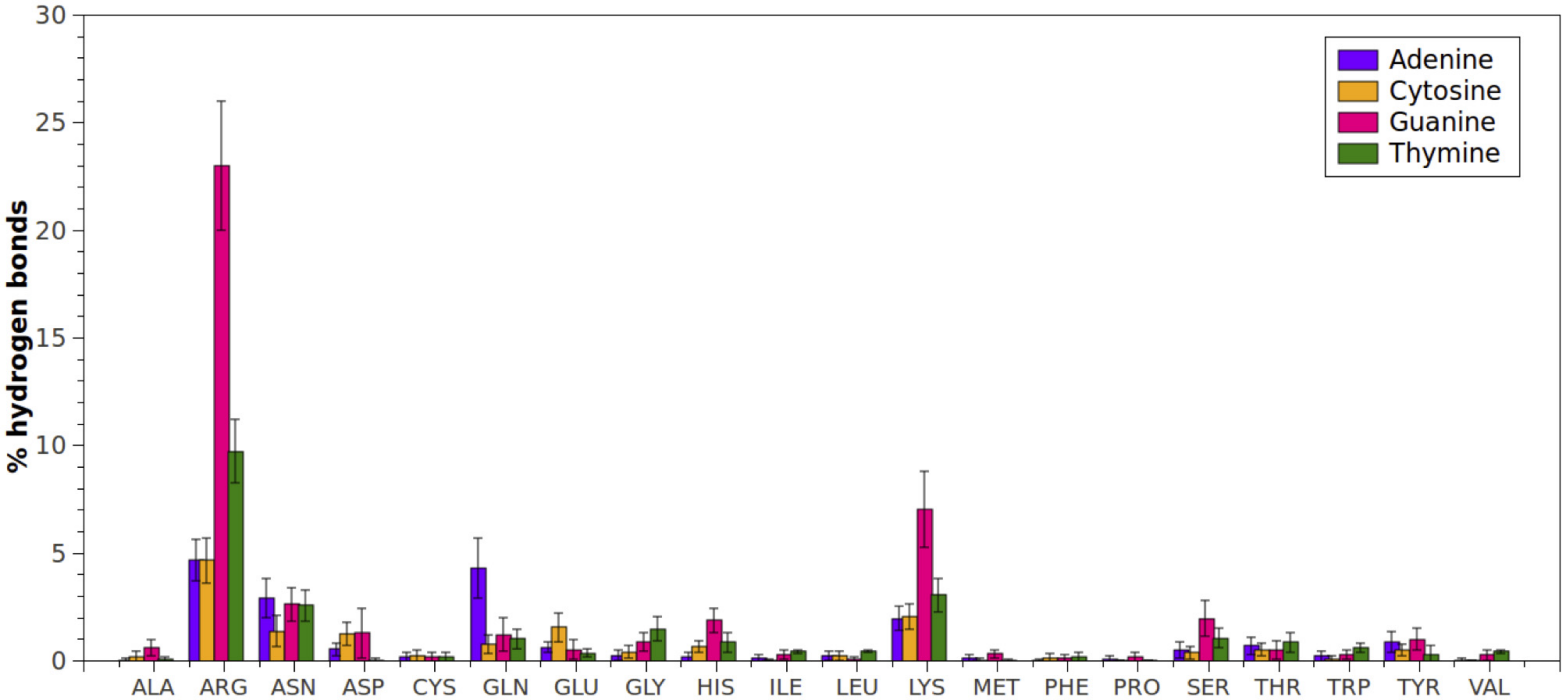
Hydrogen-bond interactions (expressed in percentages) established between amino acid type and each DNA base (Adenine, Guanine, Cytosine, Thymine). Percentages were normalized considering the total number of hydrogen-bond interactions established only with bases found in protein-dsDNA complexes.

**Fig 4:**
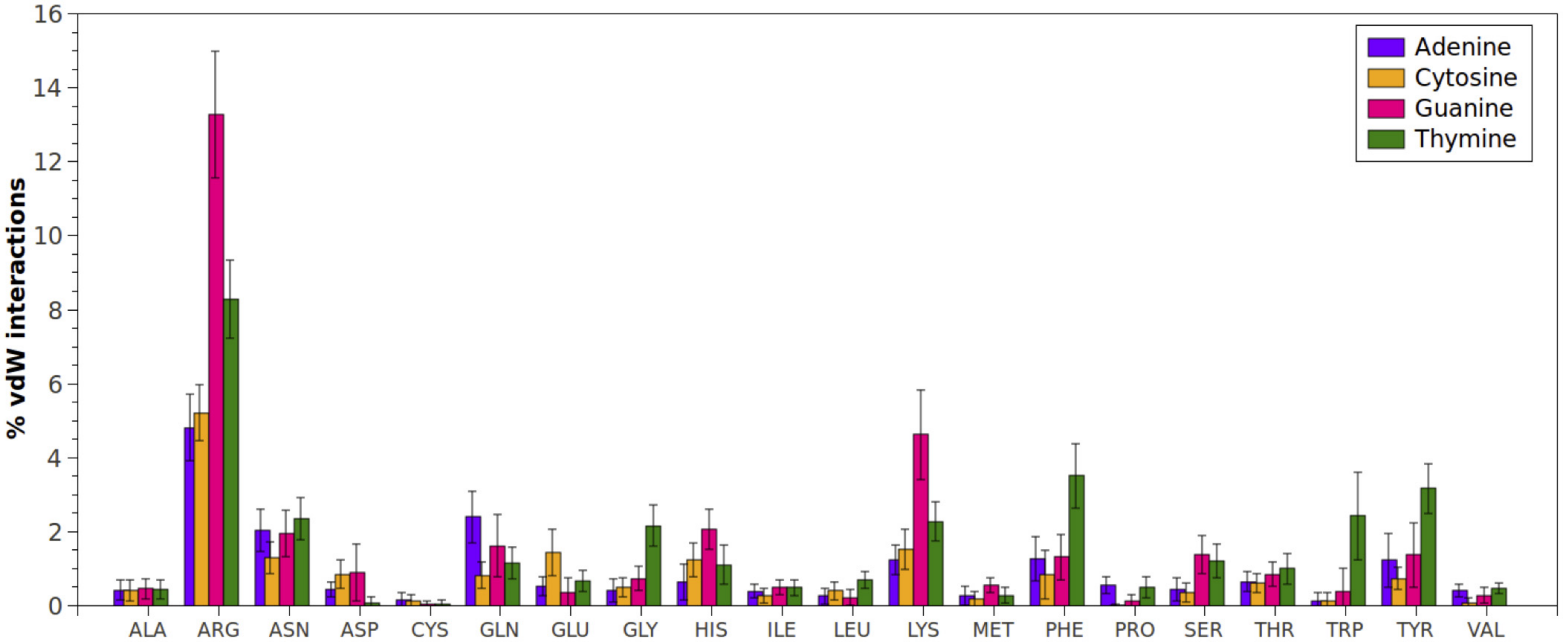
van der Waals interactions (expressed in percentages) established between amino acid types and each DNA base (Adenine, Guanine, Cytosine, Thymine). Percentages were normalized considering the total number of van der Waals interactions established only with bases found in protein-dsDNA complexes.

When the interactions between amino acids and the nitrogenous bases were analyzed (Figs 3 and 4), again Arginine and Lysine showed the highest amount of contacts. As mentioned above, at physiological pH both amino acids have terminal protonated amino groups, which make them excellent hydrogen-bond donor groups compared to other amino acids. Besides, Arginine has a guanidine terminal group that significantly increases the possibilities of generating hydrogen-bond interactions with the acceptor groups of DNA bases. vdW interactions between Arginine and bases are also relevant, since it has the ability to orient over the center of aromatic side chains in a planar stacking, which has been previously described in proteins[42].

**Fig 5:**
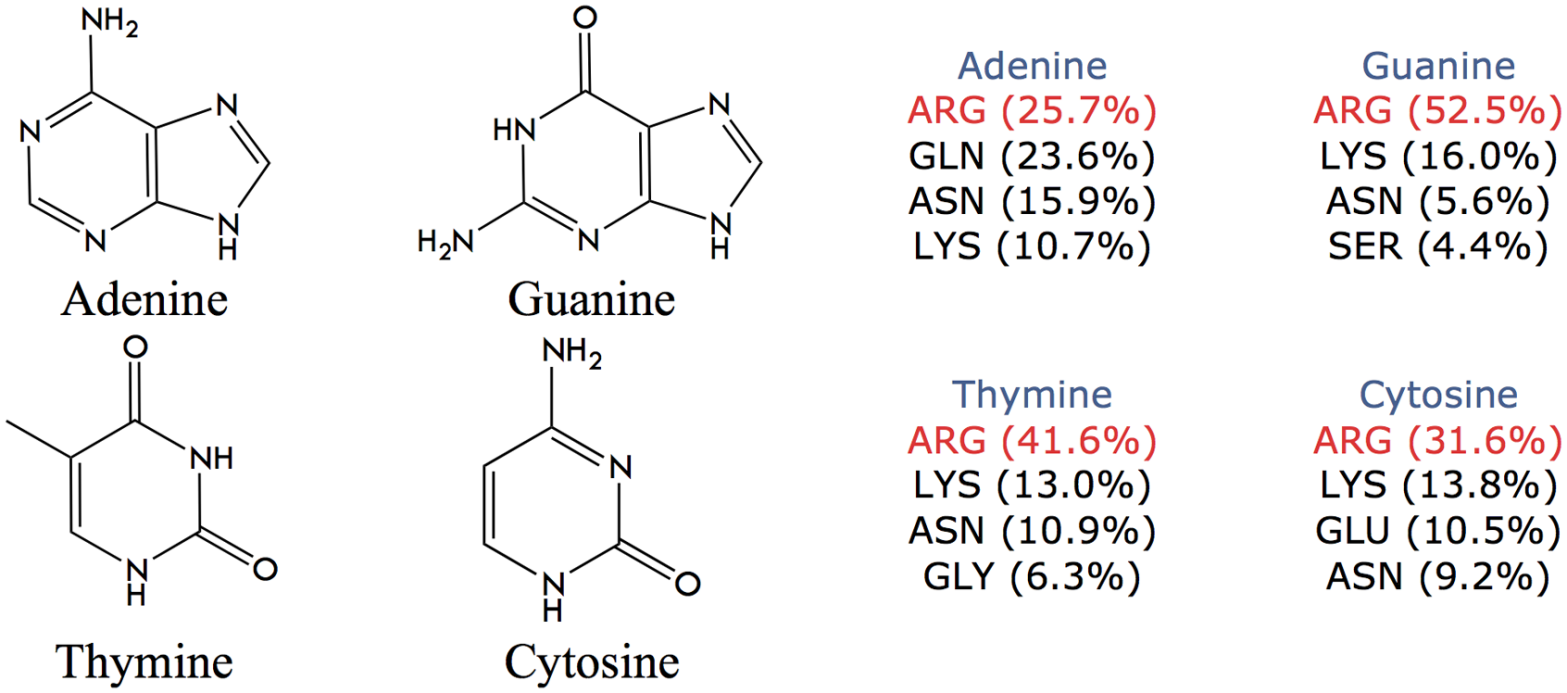
Summary of the contribution of certain amino acids to the hydrogen-bond interactions with nitrogenous bases.

The contribution of the most represented amino acids to hydrogen-bond interactions with each nitrogenous base is summarized in Fig 5. Arginine is the preferred amino acid for interaction with nitrogenous bases followed by Lysine. Interestingly, Asparagine and Glutamine also show a relevant contribution in the interaction with every nitrogenous base (Fig 5) through their amide groups, which have the property of being both donor and acceptor of H bonds at the same time. Another neutral groups with high frequency of interaction with DNA are Serine and Threonine, which together with Asparagine and Glutamine appear as new terminal groups to be considered for the functionalization of dendrimers.

Regarding the percentage of hydrogen-bond interactions established between amino acids and DNA bases, the following tendency was observed: G (10%) > T (5.3%) > A (4.1%) > C (3.4%). This trend could be associated to the number of acceptor and donor sites that each nitrogenous base has. In the case of double helix DNA, as illustrated in Fig A in S1 File, there are specific and clearly identified acceptor and donor sites after base pairing, which are available to generate hydrogen-bond interactions. Taking into account each of the possible hydrogen-bond interaction sites (Table B in S1 File), the nitrogenous base with greater number of acceptors and donors is guanine, followed by thymine and adenine, and finally cytosine (G > T = A > C). This trend agrees with the observed number of total interactions between each base and amino acids in protein-dsDNA complexes.

Arginine and Lysine, amino acids with the highest number of hydrogen-bond interactions with nitrogenous bases, exhibited the greatest interaction with guanine (Fig 3), which has the highest amount of acceptor and donor sites within its structure. On the other hand, due to the distribution of acceptors at guanine, Arginine can act as bidentate ligand[16] as Fig B in S1 File shows, increasing the number of interactions per contact. Lysine also displays this capacity, but compared to Arginine, has lower number of side chain effective configurations that can yield hydrogen-bond interactions.

Finally, when total interactions were considered, i.e. the total amount of interactions between phosphate group, sugar and bases with each amino acid (Fig C in S1 File and Fig D in S1 File), the following preference trend was identified for the interaction between dsDNA and proteins: Arginine>Lysine>Serine>Threonine. As already mentioned, Arginine and Lysine have positively-charged side chains that support the interaction with phosphate and acceptor groups belonging to the nitrogenous bases. Serine and Threonine are nucleophilic amino acids, which specifically have alcohol terminal groups and therefore, can easily interact with donor and acceptor groups in dsDNA. As neutral groups, another relevant property of Serine and Threonine is that they avoid the increase in the zeta potential of dendrimers, which is highly beneficial in the design of safer carriers.

Considering the information obtained from this analysis, it appears evident that the first amino acid to consider as terminal group in PAMAM dendrimers is Arginine. This amino acid is the best adapted to establish interactions with DNA and especially with nucleotide bases. Lysine comes up in second place; its amine side chain is already present in commercial PAMAM dendrimers. These amino acids not only posses high propensity in Protein-DNA contact zones, but are also abundant in cell-penetrating peptides which are prone to cross biological membranes. From these results it is possible to explain why dendrimers bearing these amino acids exhibit suitable performance as gene carriers, in some cases giving even better results than commercial agents[9–12]. In order to gain deeper insights at atomic level of the interaction of functionalized dendrimers with DNA, molecular simulations were performed with PAMAM-Lys and PAMAM-Arg systems, as described in the next Section of this work.

### Molecular dynamics results

PAMAM generation four (G4) was selected as the base-dendrimer to perform the amino acid functionalization, because of its lower cytotoxicity in comparison to other larger generation amino-terminated PAMAM dendrimers[43]. Considering the bioinformatics analysis performed previously in this work, and the positive experimental evidence in the application of amino acid-terminated dendrimers as gene carriers, molecular dynamics studies were performed to gain insights about structural features involved in the formation of dendrimer-DNA complexes. This kind of simulations can reveal key information about the influence of the type of amino acid conjugated to the dendrimer surface on the affinity for nucleic acids. Two PAMAM dendrimers of generation 4 were computationally modeled. Each hyper-branched polymer was modified with the following terminal groups: 64 Arginine and 64 Lysine.

#### Center of mass (COM) distance as reaction coordinate of DNA-dendrimer complexation

**Fig 6:**
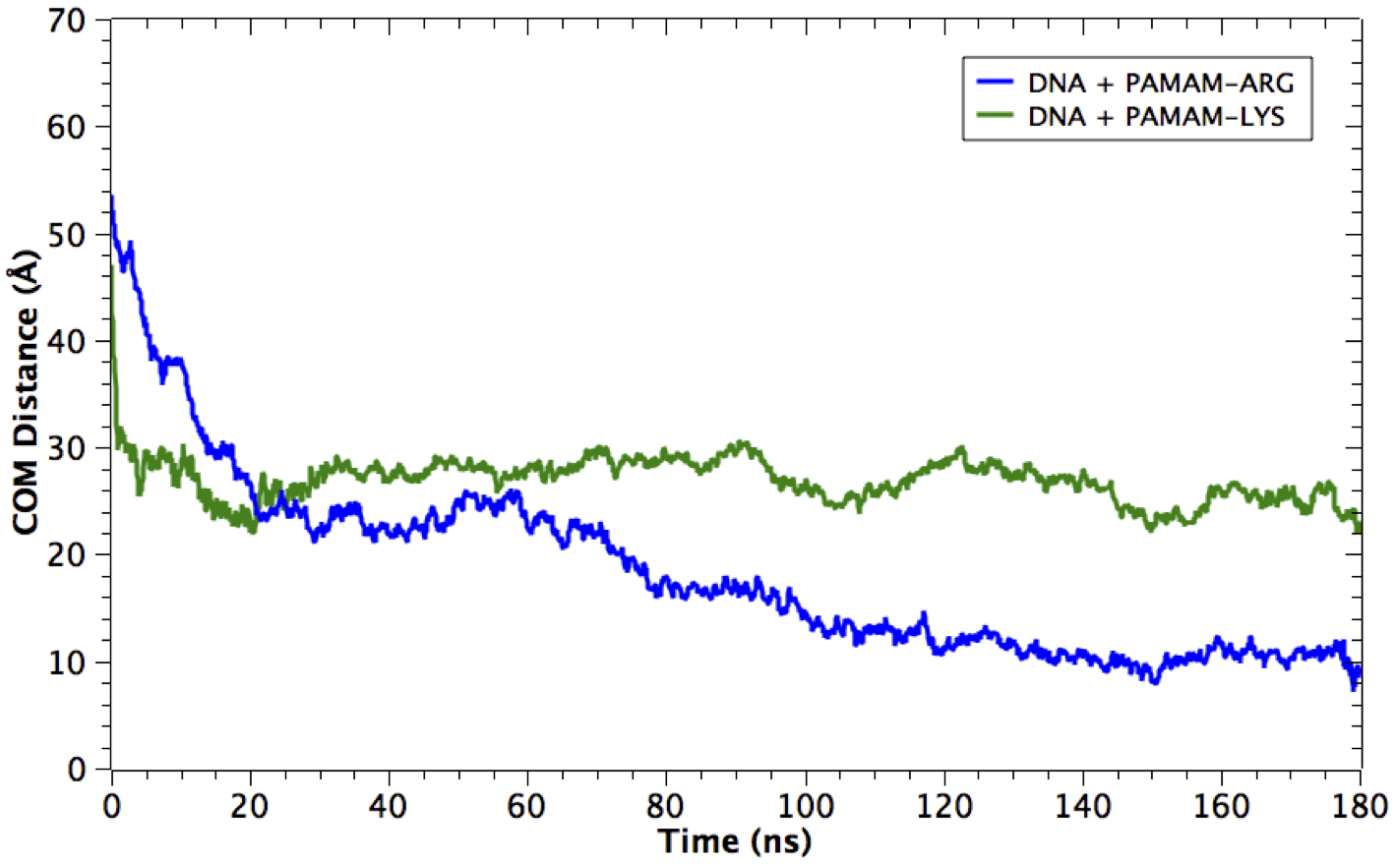
Distance between center of mass (COM) of DNA and dendrimers as a function of time. PAMAM-Arg shows a shorter distance to DNA towards the end of MD simulations.

COM distance between dsDNA and dendrimer is a metric we can use to evaluate the formation of a complex between these two molecules. Fig 6 displays the evolution of COM between dsDNA and each dendrimer over the whole MD simulations (180 ns). Starting from an initial distance of 50 Å, dendrimer and DNA progressively approach each other. It is worth noting that at the end of the trajectory the proximity reached by DNA and PAMAM-Arg is around 15 Å closer than that reached by DNA and PAMAM-Lys.

One interesting observation is that both dendrimers, PAMAM-Arg and PAMAM-Lys, reached a first stabilization distance of 25 Å from DNA at, equal to equal to the radner Coordenada de Reaccion puede generar anticuerpos.~20 ns. This suggests that this distance, equal to the radius of gyration of the dendrimers, represents the distance of first contact between the positive charges of the terminal groups of the dendrimer and the phosphate negative groups of the DNA-backbone, meanwhile the contact with the nitrogenous bases in DNA is minimum. At this first stage, probably dendrimers are not wrapping the DNA, considering that both macromolecules are kept at a distance quite similar to the radius of gyration of the dendrimer (25 Å).

Additionally, PAMAM-Lys approaches DNA faster than PAMAM-Arg but then, the dendrimer keeps a stable distance (~ 25 Å) from the nucleic acid, showing no progress in its binding dynamics during the rest of the trajectory. On the other hand, after the first 20 ns, the distance of PAMAM-Arg to DNA continues to decrease, becoming stable at around 100 ns at a distance of 10 Å. This fact means that the DNA is able to penetrate PAMAM-Arg dendrimer cavities, in contrast to PAMAM-Lys, increasing the number of contacts with this carrier.

#### Radius of gyration

**Fig 7:**
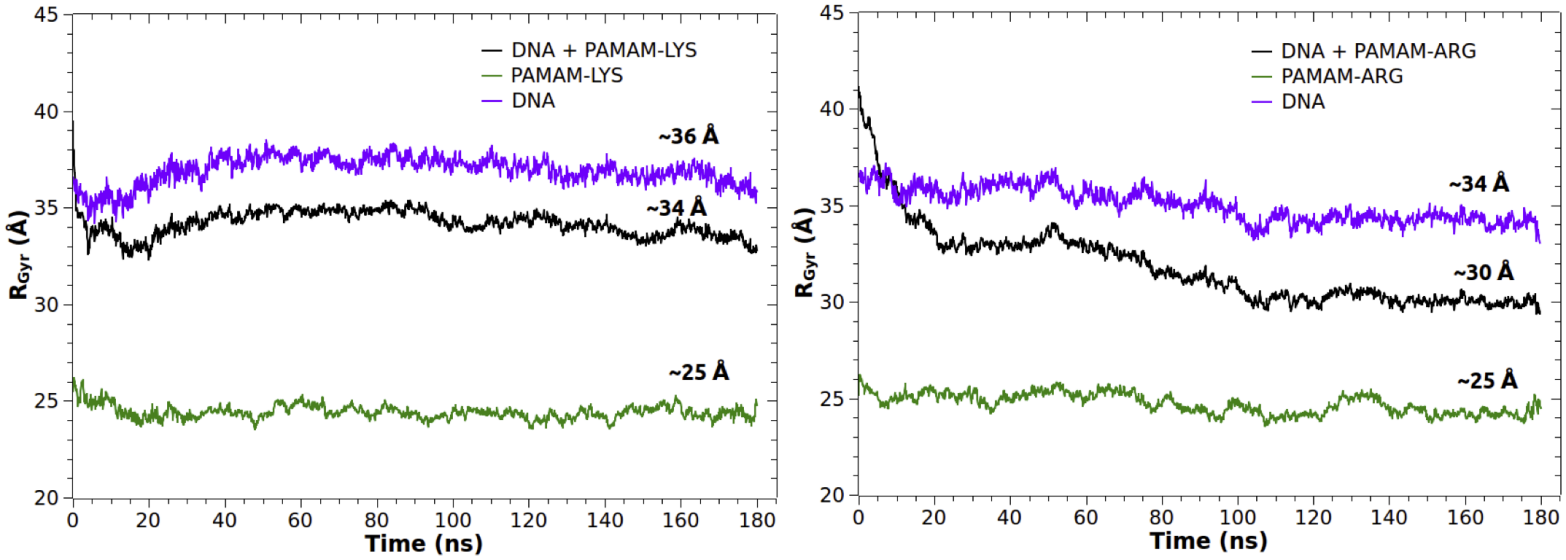
Time evolution of the radius of gyration (R_gyr_) of DNA, dendrimer and the dendrimer-DNA complex for a) PAMAM-Lys and b) PAMAM-Arg.

Radius of gyration (R_gyr_) represents the mean distance between each atom and the center of mass of a molecule. R_gyr_ is a useful value in molecular dynamics simulations to provide a structural measure of the degree of compaction of a dendrimer-DNA complex[44]. Fig 7 depicts the evolution of the radius of gyration (R_gyr_) of DNA, the dendrimer and the complex (DNA + dendrimer) for the complexation with PAMAM-Lys and PAMAM-Arg, over the full MD trajectories. The DNA displays a decrease of its radius of gyration, being more noticeable in the complex with PAMAM-Arg (reaching a R_gyr_ near 34 Å) compared to PAMAM-Lys (around 36 Å).

**Fig 8:**
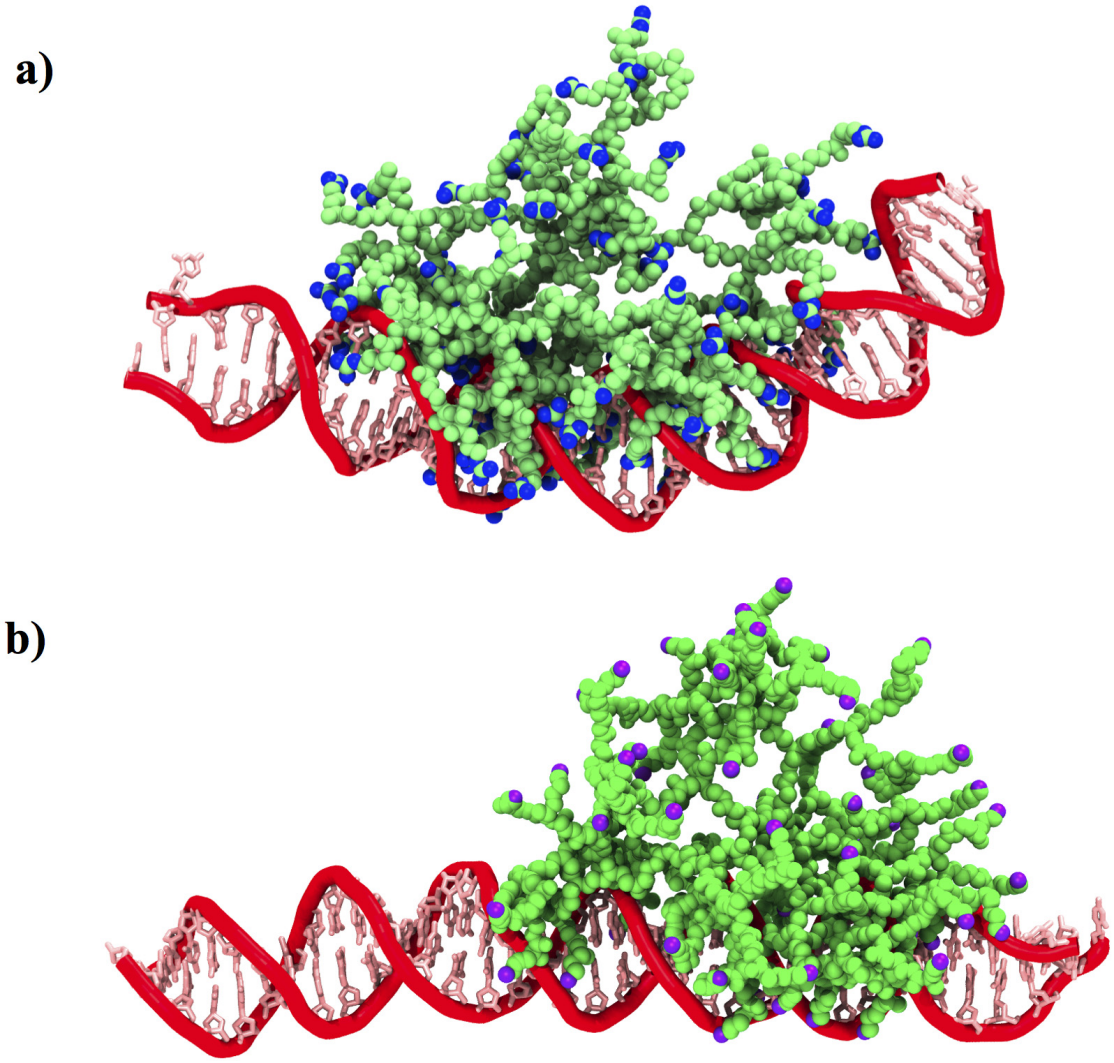
Snapshots of the last conformation of Dendrimer- DNA complexes, taken at 180 ns of MD simulations. a) PAMAM-Arg and b) PAMAM-Lys.

The radius of gyration of both dendrimers remains remarkably stable over time (~ 25 Å), suggesting that both dendrimers do not need to expand to interact with DNA. Finally, the complex PAMAM-Arg and DNA reached a more compact final structure in comparison to PAMAM-Lys / DNA, as could be distinguished from R_gyr_ values in the last fragment of the trajectory (~30 Å vs ~34 Å). Fig 8 clearly evidences the more compact and bent structure formed by PAMAM-Arg and DNA, unlike the behavior shown by PAMAM-Lys and DNA, where the DNA double strand adopted a lineal shape next to the dendrimer. This fact is due to the higher number of contacts that PAMAM-Arg is able to establish with DNA (442.7±5.3 average contacts) versus PAMAM-Lys (421.2±4.5 average contacts), generating more interaction points that finally affect the geometry of the complex.

#### Radial distribution function

The radial distribution function of dendrimers, DNA, water, sodium and chloride ions was calculated with respect to the center of mass of the dendrimers (Fig E in S1 File). The first observation is wherever the dendrimer was, the peak of the DNA radial distribution appeared when DNA reached the end terminal groups of the dendrimer, near the value of the radius of gyration of the dendrimer. However, the distribution in this zone is wider for PAMAM-Arg, suggesting that the penetration of the DNA into this dendrimer is larger. This result is in agreement with the center of mass distance displayed in Fig 6.

Meanwhile, chloride ions form a stable solvation shell around the Dendrimer-DNA complex in both complexes, with their radial distribution falling close to the water density towards the radius of gyration of each complex. Here the water and ions penetrate the core of both dendrimers (~ 5 Å) and the resulting swelling appears higher in the case of PAMAM-Lys, which is reflected in a water density value close to 1 in the proximities of the core. This observation also supports the notion of a wider and more rigid conformation of PAMAM-Lys compared to PAMAM-Arg. In consequence, the PAMAM-Arg system seems to be more “adaptable” to the DNA strand. In fact, the water density fell to values lower than 1 in the internal cavities of PAMAM-Arg, due to the higher penetration of the DNA strand in comparison with PAMAM-Lys.

#### Structural properties of DNA in complex with dendrimers

Even if the size of the dendrimers, evidenced from R_gyr_ calculations, is not affected by the binding with DNA, as previous articles have shown regarding PAMAM dendrimers, it is important to determine the deformability degree of these nanoparticles in the presence of charged ligands such as nucleic acids. The wrapping of DNA around the charged surface of the dendrimer has been described as similar to its condensation into histone proteins, which like the dendrimers, are positively charged macromolecules. In fact, the globular structure of dendrimers (especially at higher generations) has been frequently compared with that of proteins[45].

To study the capacity of these dendrimers to wrap nucleic acids, the degree of shortening of the DNA was determined over the entire MD trajectory (Fig F in S1 File). As can be clearly observed, PAMAM-Arg is able to bend DNA to a higher extent than PAMAM-Lys (~25% vs only ~5%, respectively). In spite of this difference, both dendrimers are able to protect DNA from water as revealed from surface accessible solvent area (SASA) of the DNA in complex with PAMAM-Arg and PAMAM-Lys (Fig F in S1 File). This analysis shows the value of SASA of the DNA alone in solvent and how the complexation with dendrimers causes a decrease of the solvent accessible area of the DNA in a similar degree for both PAMAM-Arg and PAMAM-Lys.

#### Free energy of binding

As already mentioned, MM-GB/PBSA has been broadly implemented to study energetics involved in complexation of DNA-dendrimer systems, despite its limitations associated with the use of a continuum model for the solvent, SASA as a descriptor of hydrophobic effect, and a poor estimation of the entropic term. Nevertheless, MM-GB/PBSA offers successful results in predicting the behavior of several systems including ligand affinity to proteins.

The high contribution of the electrostatic energy to the binding energy between dendrimers and DNA has been discussed in many articles. Nandy and Maiti[25] described affinity for DNA using dendrimers of different generations with the MM-GBSA method, demonstrating that a dendrimer with a higher number of charges such PAMAM G5, could associate with DNA with better affinity than PAMAM G4 or PAMAM G3. However, PAMAM G4 appeared, according to the discussion of the authors, as a better candidate for DNA transfection than G5 due to structural aspects such as flexibility.

In the present study, the number of charges of both dendrimers PAMAM-Arg and PAMAM-Lys is the same, so an *a priori* idea could be that both of them bind DNA with similar affinity. However, as Table 2 shows, PAMAM-Arginine is by far the dendrimer with higher affinity for DNA (-282.24 kcal/mol vs -87.66 kcal/mol for PAMAM-Lys). Thus, the introduction of Arginine or specifically the guanidine group is playing a key role in the binding of nucleic acids, mainly due to the increased number of contacts introduced by bidentate interactions with nucleotide bases.

**Table 2:** MM-GBSA Binding Free energy results.

Complex	ΔH_bind_(kcal/mol)	-TΔS (kcal/mol)	ΔG_bind_ (kcal/mol)
G4-Arg + DNA	-348.58 ± 4.3	66.34	-282.24
G4-Lys + DNA	-153.69 ± 9.2	66.03	-87.66

#### Contribution of DNA groups to the interaction with amino acid end groups

**Fig 9:**
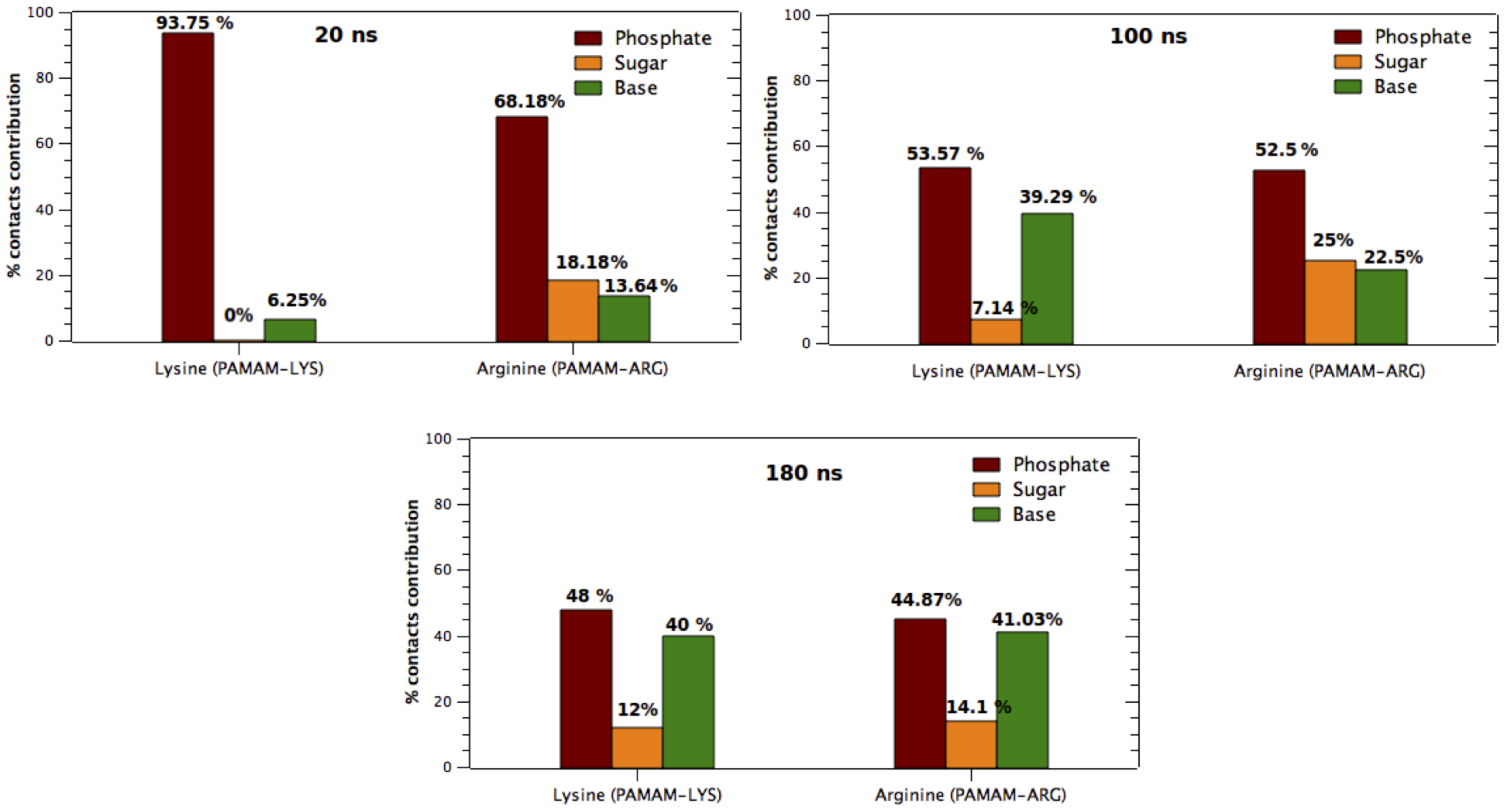
Contacts (%) of Lysine and Arginine terminal groups of dendrimers with phosphate, deoxyribose and bases of the DNA, obtained from three snapshots of MD simulations (20, 100 and 180 ns) of dendrimer-dsDNA complexes.

To provide insight in determining if at the first stage of molecular simulation the dendrimer—DNA interaction is being driven mainly through the phosphate groups, the total number of contacts between each DNA group (sugar, phosphate and bases) and terminal groups of the dendrimers, Arginine or Lysine, were obtained along the simulations, to determine the percentage contribution of each interaction. From the results shown in Fig 9, it appears evident from the first 20 ns that the predominant interaction is mainly dominated by the contacts of the terminal groups (Lys/Arg) and DNA phosphate groups.

It can be distinguished that the phosphate contribution decays over the MD trajectory, as the contacts to nitrogenous bases and sugar groups increases. Results obtained towards the end of the simulation, when both DNA-dendrimer complexes have reached convergence (according to COM results), are in agreement with the evidence observed from studies of Protein-DNA complexes, which demonstrated that the number of interactions between amino acids and DNA groups follows the order Phosphate > Bases > Sugar.

#### Contribution of DNA bases to the interaction with amino acid end groups

**Fig 10:**
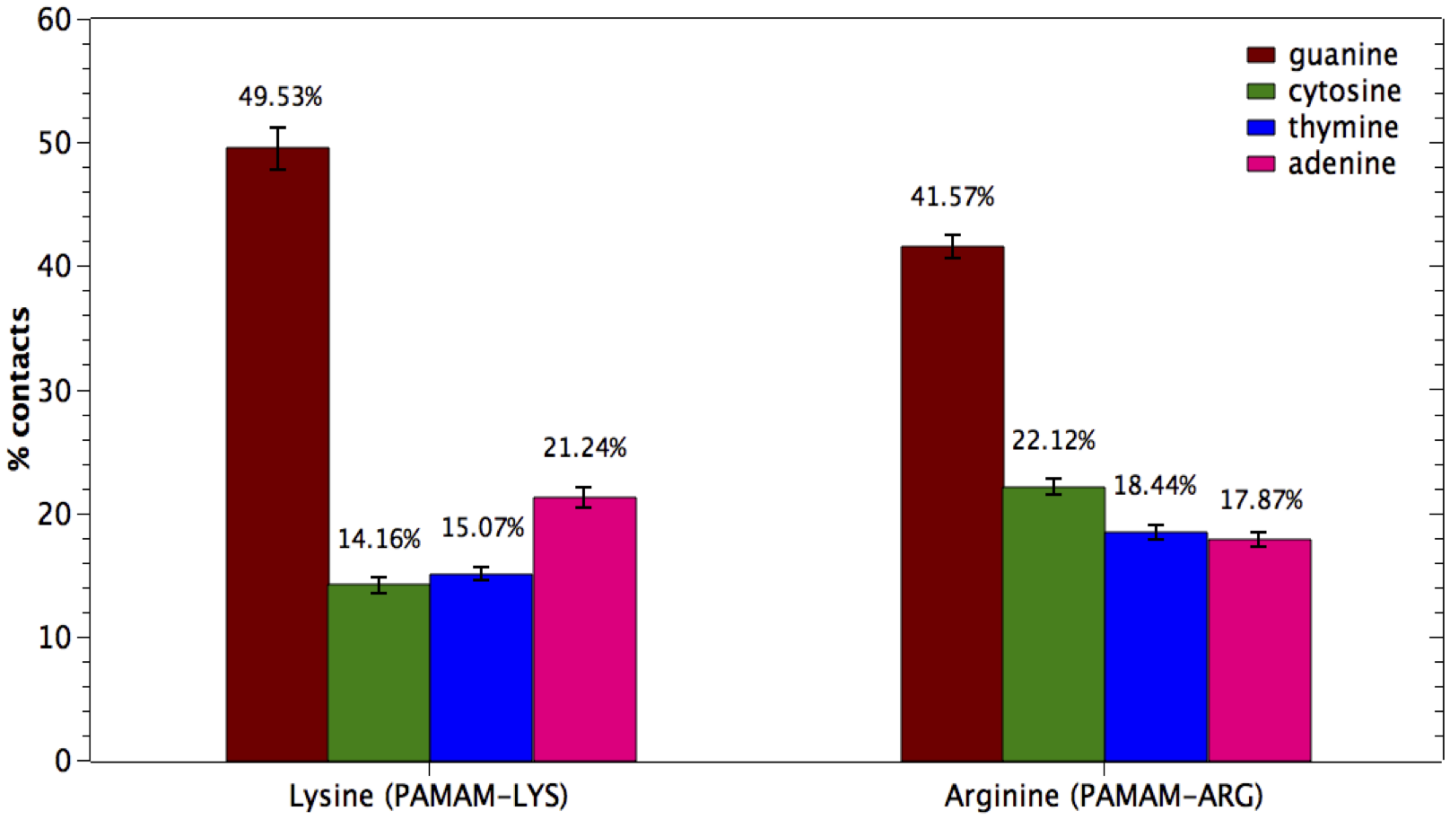
Contribution of each nitrogenous base to the interaction with Arginine/Lysine terminal groups of the dendrimers. 100% corresponds to the total number of contacts established only with bases of DNA (not including interaction with sugar or phosphate groups).

The contribution of each DNA base (Adenine, Cytosine, Guanine, Thymine) to the interaction with Arginine and Lysine end-groups of the dendrimers was studied. The number of contacts between Arginine/Lysine groups involved in the interaction with each base, within a cutoff of 3.5 Å, was obtained for the last 40 ns of trajectory for each system and shown in Fig 10. Interestingly, Guanine retains a relevant role in the interaction with Arginine and Lysine, accounting for about ~49% and 41% of the contacts, respectively. Meanwhile, the contributions of the other bases are similar to each other, reaching values around 15%- 22%. These data are in agreement with the Nucleic-Acid Database analysis shown in the previous section of this work, where a key role of Guanine in the interaction with Arginine residues was demonstrated for proteins in complex with DNA.

## Future Considerations

Previous articles have addressed the functionalization of dendrimers with different amino acids to generate new gene carriers[15]. Some studies have shown the functionalization of dendrimer-terminal groups with Arginine and Lysine[13, 46–48], as a way to obtain improved protonated DNA carriers. One of these articles published by Choi et al.[9] revealed that PAMAM-Arginine dendrimers of generation four have improved transfection efficiency over PAMAM G4-Lysine (about ~ 25% more efficient) and native PAMAM G4, but have higher cytotoxicity. Dendrimers conjugated with Arginine or Lysine have more positive charges than a native dendrimer of the same generation due to the charged amino groups belonging to the main chain of the amino acid, in addition to the side chain group. This condition increases the zeta potential, and could be the reason of the higher cytotoxicity. Furthermore, dendrimer flexibility allows induction of distortion in the DNA structure which has been described as an important parameter of a gene carrier[25]. In this sense, the highly charged surface of PAMAM-Arg or PAMAM-Lys (two-fold increase over native PAMAM G4) generates a wider and a more rigid structure, as a result of the repulsion of charged terminal groups.

Whereas high generation and highly cationic dendrimers have been described to induce cytotoxicity, the neutralization of some terminal groups has been proposed to address this inconvenient[7]. Thus, both situations mentioned above may be avoided if dendrimers are conjugated only to the side chains of the positively charged amino acids, or conjugating them to non-charged amino acids with high affinity for DNA. Our bioinformatics analyses suggest that the residues that most commonly interact with DNA are basic amino acids, yet other residues with neutral side-chain groups also contribute to modulate the protein-DNA interaction in different cellular events.

Another relevant aspect to consider in the dendrimer design process of nucleic acid carriers is the balance between the size of the nanoparticle and the number of positive surface charges—or density of charges. In order to determine the density of charges as a function of the surface area of each protein, one of the 493 protein-DNA subsets generated before was selected, and inspected using the following scheme. First, the radius of gyration of each protein (biological assembly) was calculated using the VMD software. As a coarse approach, the surface area of each protein was calculated assuming spherical dimensions ~ 4πr^2^. Next, the degree of solvent accessibility for each protein residue was estimated using GetArea (http://curie.utmb.edu/getarea.html)[49, 50]. This server provides the percentage of solvent accessibility of each residue with respect to the solvent accessible surface area of a particular amino acid in an extended peptide. Thus, a SASA percentage higher than 50% means that the amino acid is exposed to the solvent. In this way, we obtained the SASA percentage for every residue of each protein (biological assembly) of the subset, to determine the number of amino acids of a certain type (positive, negative or neutral) effectively exposed to the solvent. Finally, the ratio between the total number of exposed residues of each type and the spherical surface area of each protein was calculated as follows (Fig G in S1 File):

$$Density\;of\;charges=\;\frac{Number\;of\;charges\;\left(e\right)}{Surface\;area\;\left(nm^{2}\right)}$$

From the analysis of protein-DNA complexes, the density value of positive and negative charges is almost the same (~0.4), whereas the number of neutral residues exposed to the solvent is higher (~0.9).

The same analysis was then applied on the Lysine/Arginine dendrimers. The estimated spherical surface area for both systems is 78.50 nm^2^ (radius of gyration ~ 2.5 nm), and since both of them are totally functionalized with positively charged groups, their density of charges corresponds to 1.6 (128*e* / 78.50 nm^2^). This value is by far higher than that found in proteins binding DNA (~ 0.4) suggesting that the number of positive charges in the surface of these proteins does not need to be too high to be able to condense nucleic acids. Following this observation, one can consider the mean density of positive charges for proteins and then design a dendrimer of the same size of the ones described here, but only considering 25% of functionalization with arginine groups. The rest of the available terminal sites of the dendrimer could be functionalized with neutral moieties with high propensity to bind DNA, as we suggested above.

## Conclusion

Biomimetic tools made possible to explain experimental evidence related to better DNA delivery performance of dendrimers modified with two amino acids: Arginine and Lysine. Natural abundance of interactions between amino acids and dsDNA gave insights about the relevance of the interactions to phosphate and sugar, and also allowed us to identify those amino acids (Arg and Lys) with the highest propensity of being part of the DNA-protein contact zone, agreeing with previously reported experimental results. Molecular dynamics simulations explain the higher affinity of the PAMAM-Arg system for DNA in comparison to PAMAM-Lys and delivered key information about the interaction of these dendrimers with DNA at the atomic level.

From the analysis described above, it appears consistent that the conjugation of dendrimers with amino acid residues gives these nanoparticles typical features of proteins, mainly related with the specificity in the interaction with certain chemical groups, in this case, DNA sugar, phosphate and bases. By studying structural data of proteins and their complexes with several kinds of ligands, it is possible to determine new functional groups for inclusion in dendrimers, allowing to transform these hyper-branched polymers into tunable nanoparticles with affinity for some specific target.

## Supporting Information

S1 File **Table A**, Percentage of hydrogen bonds and vdW interactions between amino acids and oligonucleotides in non-homologous sets of protein-dsDNA complexes; **Table B**, Number of acceptor and donor sites for each base pair A-T and C-G in dsDNA; **Fig A**, Schematic representation of the acceptor and donor sites available for hydrogen-bond interactions in base pairs thymine-adenine and cytosine-guanine; **Fig B**, Schematic diagram of the bidentate interactions between the amino acids arginine and lysine, and guanine. (Adapted from Luscombe et al.^11^); **Fig C**, Total hydrogen-bond interactions (expressed in percentages) between each amino acid and phosphate groups, nitrogenous bases and deoxyribose found in protein-dsDNA complexes; **Fig D**, Total vdW interactions (expressed in percentages) between each amino acid and phosphate groups, nitrogenous bases and deoxyribose found in protein-dsDNA complexes; **Fig E**, Radial distribution function of DNA, the dendrimer, water and counterions (Na^+^/Cl^-^) in each complex. a) PAMAM-Arg and b) PAMAM-Lys. The distribution was calculated with respect to the center of mass of the dendrimer; **Fig F**, a) Solvent accessible surface area (SASA) of dsDNA alone in solution (black line), in complex with PAMAM-Arg (blue line) and PAMAM-Lys (green line).b) dsDNA shortening (%) as a function of time in the presence of PAMAM-Arg and PAMAM-Lys; **Fig G**, Histogram of the density of charges exposed to the solvent, expressed as the ratio between the normalized number of charges and surface area (nm2). Data were obtained from a single subset of non-homologous dsDNA-protein complexes.
(DOC)
