# Supplementary material for: Biomimetics: From Bioinformatics to Rational Design of Dendrimers as Gene Carriers
Source: PLoS One. 2015 Sep 18;10(9):e0138392. doi: 10.1371/journal.pone.0138392 (PMC4575034; doi:10.1371/journal.pone.0138392)

**Supporting Information (S1 File)**

PLoS One Supporting Information

Article title: Biomimetics: From Bioinformatics to Rational Design of Dendrimers as Gene Carriers

Authors: Valeria Márquez-Miranda, María Belén Camarada, Ingrid Araya-Durán, Ignacio Varas-Concha, Daniel Eduardo Almonacid, Fernando Danilo González-Nilo.

The following Supporting Information is available for this article:

**Table A**. Percentage of hydrogen bonds and vdW interactions between amino acids and oligonucleotides in non-homologous sets of protein-dsDNA complexes.

**Table B.** Number of acceptor and donor sites for each base pair A-T and C-G in dsDNA.

**Figure A.** Schematic representation of the acceptor and donor sites available for hydrogen-bond interactions in base pairs thymine-adenine and cytosine-guanine.

**Figure B.** Schematic diagram of the bidentate interactions between the amino acids arginine and lysine, and guanine. (Adapted from Luscombe et al.^11^).

**Figure C.** Total hydrogen-bond interactions (expressed in percentages) between each amino acid and phosphate groups, nitrogenous bases and deoxyribose found in protein-dsDNA complexes.

**Figure D.** Total vdW interactions (expressed in percentages) between each amino acid and phosphate groups, nitrogenous bases and deoxyribose found in protein-dsDNA complexes.

**Figure E.** Radial distribution function of DNA, the dendrimer, water and counterions (Na^+^/Cl^-^) in each complex. a) PAMAM-Arg and b) PAMAM-Lys. The distribution was calculated with respect to the center of mass of the dendrimer

**Figure F.** a) Solvent accessible surface area (SASA) of dsDNA alone in solution (black line), in complex with PAMAM-Arg (blue line) and PAMAM-Lys (green line).b) dsDNA shortening (%) as a function of time in the presence of PAMAM-Arg and PAMAM-Lys.

**Figure G.** Histogram of the density of charges exposed to the solvent, expressed as the ratio between the normalized number of charges and surface area (nm2). Data were obtained from a single subset of non homologous dsDNA-protein complexes.

**Table A**

| **HBonds** |  |  |  |  |  |  |  |  |
| --- | --- | --- | --- | --- | --- | --- | --- | --- |
|  | **A** | **C** | **G** | **T** | **P** | **S** | **Total** | **Total–(S+P)** |
| ALA | 0.003 | 0.033 | 0.138 | 0.009 | 1.776 | 0.117 | 2.076 | 0.183 |
| ARG | 1.067 | 1.059 | 5.252 | 2.219 | 19.248 | 3.410 | 32.255 | 9.597 |
| ASN | 0.664 | 0.309 | 0.596 | 0.583 | 3.268 | 0.660 | 6.080 | 2.152 |
| ASP | 0.120 | 0.284 | 0.293 | 0.006 | 0.293 | 0.189 | 1.185 | 0.703 |
| CYS | 0.034 | 0.045 | 0.033 | 0.036 | 0.314 | 0.037 | 0.499 | 0.148 |
| GLN | 0.981 | 0.171 | 0.274 | 0.228 | 1.969 | 0.340 | 3.963 | 1.654 |
| GLU | 0.138 | 0.354 | 0.115 | 0.076 | 0.265 | 0.262 | 1.209 | 0.682 |
| GLY | 0.053 | 0.089 | 0.196 | 0.336 | 2.590 | 0.810 | 4.074 | 0.674 |
| HIS | 0.035 | 0.149 | 0.423 | 0.191 | 1.814 | 0.834 | 3.446 | 0.798 |
| ILE | 0.022 | 0.000 | 0.056 | 0.096 | 0.853 | 0.116 | 1.143 | 0.174 |
| LEU | 0.050 | 0.049 | 0.009 | 0.097 | 0.951 | 0.094 | 1.250 | 0.205 |
| LYS | 0.444 | 0.463 | 1.602 | 0.692 | 11.198 | 1.357 | 15.756 | 3.201 |
| MET | 0.021 | 0.003 | 0.068 | 0.001 | 0.222 | 0.014 | 0.329 | 0.093 |
| PHE | 0.002 | 0.027 | 0.022 | 0.034 | 0.616 | 0.025 | 0.726 | 0.085 |
| PRO | 0.013 | 0.000 | 0.036 | 0.000 | 0.000 | 0.002 | 0.051 | 0.049 |
| SER | 0.107 | 0.080 | 0.443 | 0.235 | 7.166 | 1.717 | 9.748 | 0.865 |
| THR | 0.157 | 0.114 | 0.108 | 0.191 | 7.151 | 1.508 | 9.229 | 0.570 |
| TRP | 0.046 | 0.015 | 0.060 | 0.135 | 0.974 | 0.052 | 1.282 | 0.256 |
| TYR | 0.191 | 0.111 | 0.226 | 0.066 | 3.236 | 0.748 | 4.578 | 0.594 |
| VAL | 0.005 | 0.000 | 0.055 | 0.096 | 1.481 | 0.153 | 1.790 | 0.156 |
| **Total** | 4.153 | 3.355 | 10.005 | 5.327 | 65.385 | 12.445 |  |  |

| **vdW** |  |  |  |  |  |  |  |  |
| --- | --- | --- | --- | --- | --- | --- | --- | --- |
|  | **A** | **C** | **G** | **T** | **P** | **S** | **Total** | **Total–(S+P)** |
| ALA | 0.103 | 0.104 | 0.111 | 0.109 | 1.588 | 0.500 | 2.515 | 0.427 |
| ARG | 1.208 | 1.310 | 3.339 | 2.081 | 12.927 | 6.994 | 27.859 | 7.938 |
| ASN | 0.510 | 0.324 | 0.488 | 0.589 | 2.113 | 1.409 | 5.433 | 1.911 |
| ASP | 0.108 | 0.210 | 0.220 | 0.018 | 0.500 | 0.576 | 1.632 | 0.556 |
| CYS | 0.035 | 0.027 | 0.006 | 0.008 | 0.178 | 0.039 | 0.293 | 0.076 |
| GLN | 0.602 | 0.203 | 0.403 | 0.286 | 1.484 | 1.183 | 4.161 | 1.494 |
| GLU | 0.130 | 0.362 | 0.089 | 0.162 | 0.572 | 0.567 | 1.882 | 0.743 |
| GLY | 0.101 | 0.123 | 0.181 | 0.542 | 2.587 | 1.856 | 5.390 | 0.947 |
| HIS | 0.159 | 0.307 | 0.517 | 0.275 | 1.404 | 1.056 | 3.718 | 1.258 |
| ILE | 0.095 | 0.066 | 0.121 | 0.121 | 0.959 | 0.722 | 2.084 | 0.403 |
| LEU | 0.061 | 0.100 | 0.053 | 0.174 | 0.801 | 0.389 | 1.578 | 0.388 |
| LYS | 0.310 | 0.381 | 1.161 | 0.570 | 8.794 | 2.020 | 13.236 | 2.422 |
| MET | 0.065 | 0.041 | 0.136 | 0.067 | 0.276 | 0.318 | 0.903 | 0.309 |
| PHE | 0.318 | 0.209 | 0.328 | 0.882 | 0.790 | 0.845 | 3.372 | 1.737 |
| PRO | 0.135 | 0.000 | 0.027 | 0.124 | 0.606 | 0.335 | 1.227 | 0.286 |
| SER | 0.108 | 0.086 | 0.343 | 0.303 | 4.837 | 1.686 | 7.363 | 0.840 |
| THR | 0.161 | 0.149 | 0.212 | 0.248 | 4.841 | 1.466 | 7.077 | 0.770 |
| TRP | 0.029 | 0.029 | 0.091 | 0.608 | 0.468 | 0.374 | 1.599 | 0.757 |
| TYR | 0.307 | 0.182 | 0.342 | 0.795 | 2.874 | 1.920 | 6.420 | 1.626 |
| VAL | 0.101 | 0.015 | 0.068 | 0.113 | 1.410 | 0.513 | 2.220 | 0.297 |
| **Total** | 4.646 | 4.228 | 8.236 | 8.075 | 50.009 | 24.768 |  |  |

**Table B**

|  | **Acceptors** | **Donors** | **Total** |
| --- | --- | --- | --- |
| **A** | 2 | 1 | 3 |
| **T** | 3 | 0 | 3 |
| **C** | 1 | 1 | 2 |
| **G** | 3 | 1 | 4 |

**Figure A**

|  |  |
| --- | --- |

**Figure B**

| 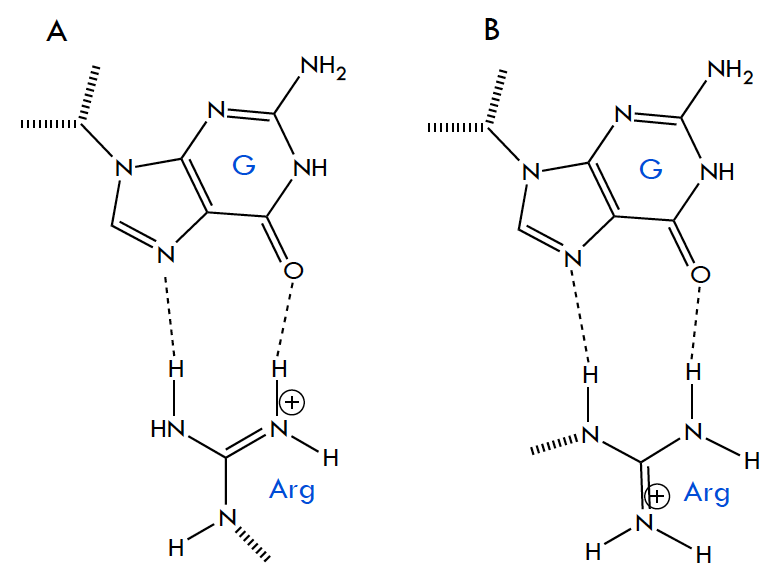 | 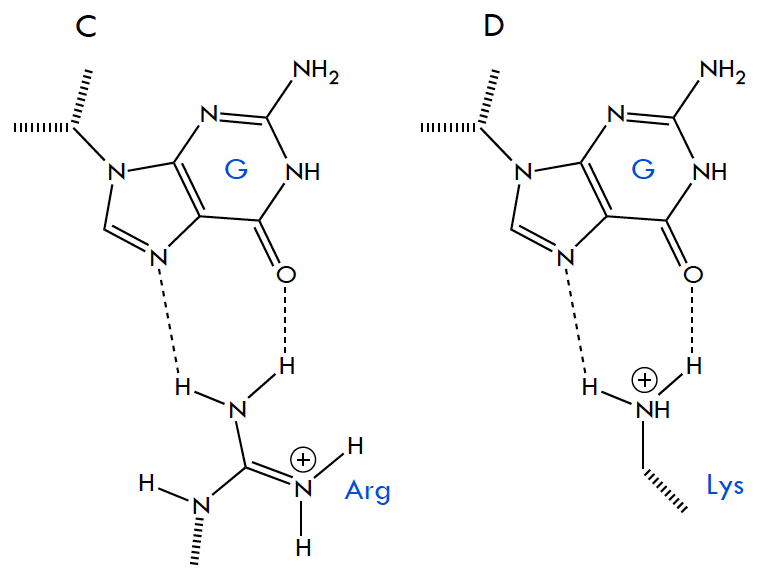 |
| --- | --- |

**Figure C**


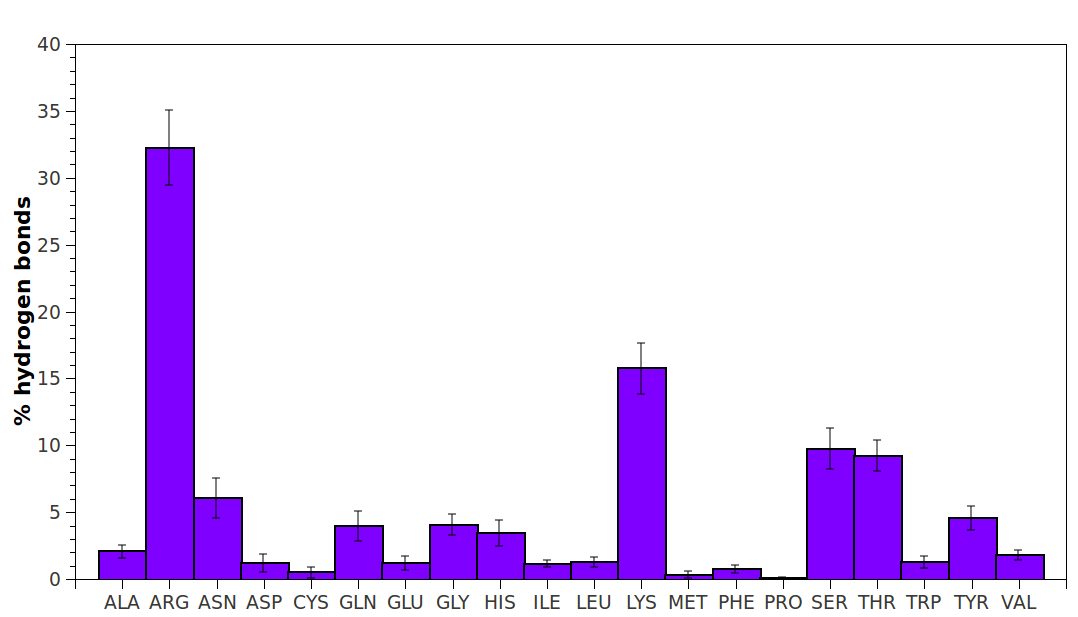


**Figure D**


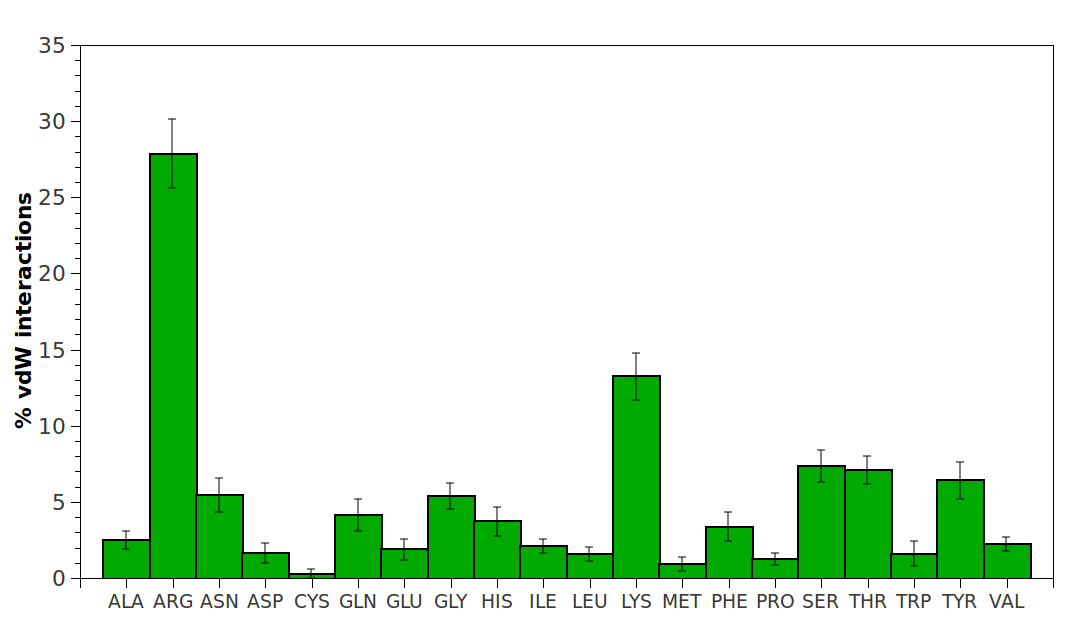


**Figure E.**


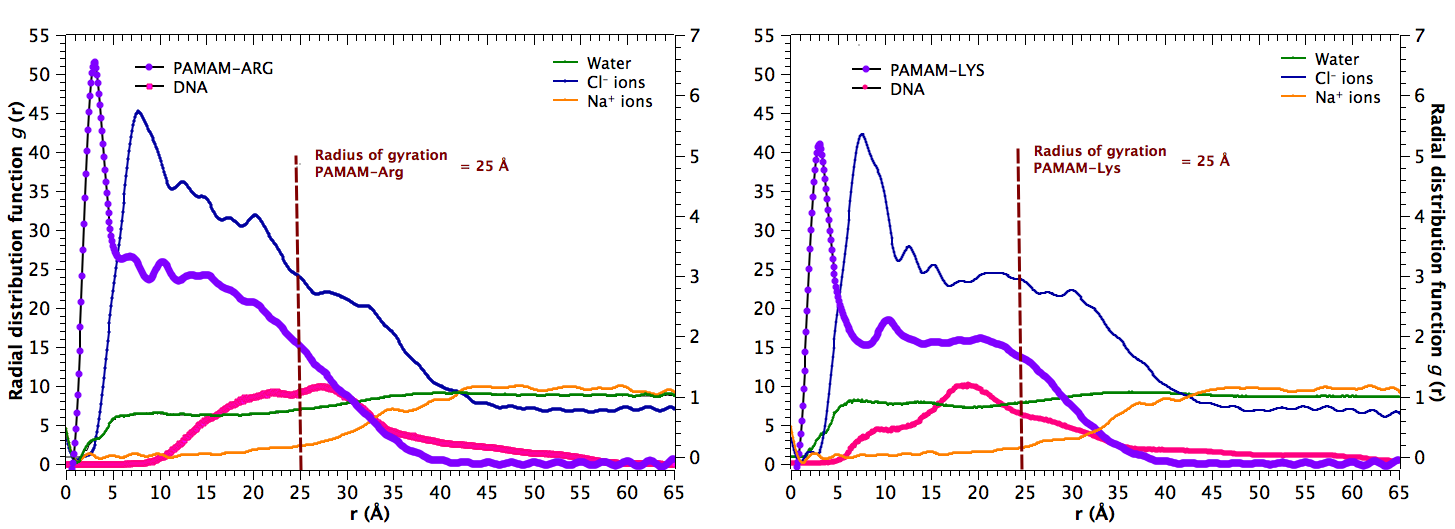


**.**

**Figure F**

**
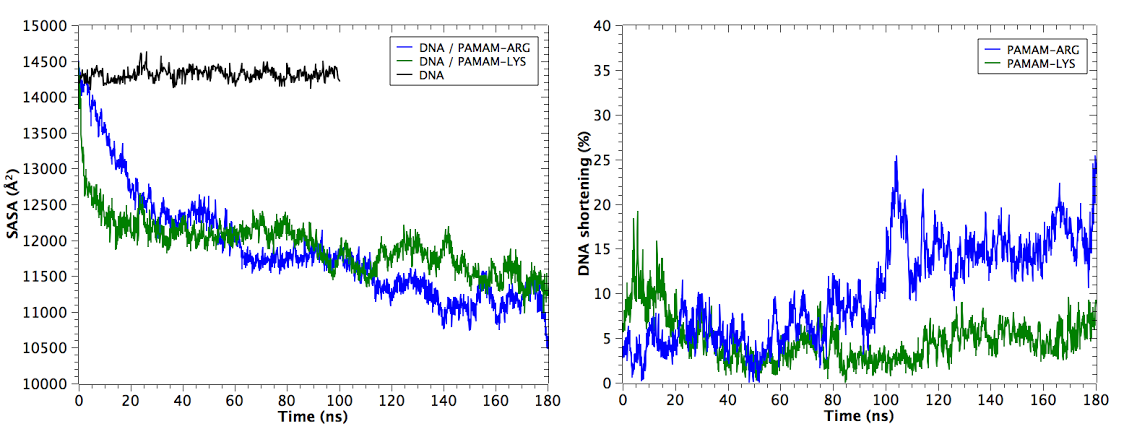
**

**Figure G**


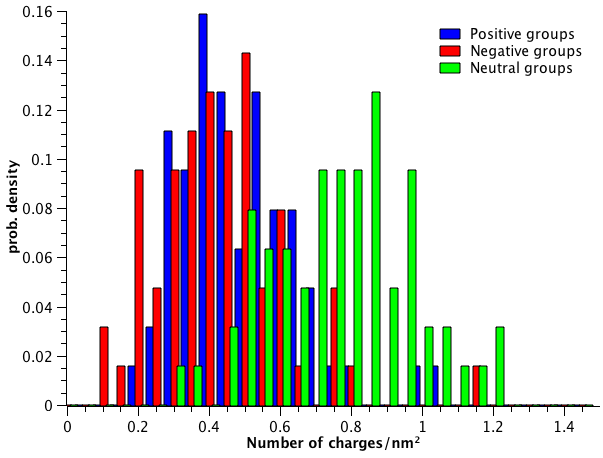

Supplement: S1 File — Table A, Percentage of hydrogen bonds and vdW interactions between amino acids and oligonucleotides in non-homologous sets of protein-dsDNA complexes; Table B, Number of acceptor and donor sites for each base pair A-T and C-G in dsDNA; Fig A, Schematic representation of the acceptor and donor sites available for hydrogen-bond interactions in base pairs thymine-adenine and cytosine-guanine; Fig B, Schematic diagram of the bidentate interactions between the amino acids arginine and lysine, and guanine. (Adapted from Luscombe et al.11); Fig C, Total hydrogen-bond interactions (expressed in percentages) between each amino acid and phosphate groups, nitrogenous bases and deoxyribose found in protein-dsDNA complexes; Fig D, Total vdW interactions (expressed in percentages) between each amino acid and phosphate groups, nitrogenous bases and deoxyribose found in protein-dsDNA complexes; Fig E, Radial distribution function of DNA, the dendrimer, water and counterions (Na+/Cl-) in each complex. a) PAMAM-Arg and b) PAMAM-Lys. The distribution was calculated with respect to the center of mass of the dendrimer; Fig F, a) Solvent accessible surface area (SASA) of dsDNA alone in solution (black line), in complex with PAMAM-Arg (blue line) and PAMAM-Lys (green line).b) dsDNA shortening (%) as a function of time in the presence of PAMAM-Arg and PAMAM-Lys; Fig G, Histogram of the density of charges exposed to the solvent, expressed as the ratio between the normalized number of charges and surface area (nm2). Data were obtained from a single subset of non-homologous dsDNA-protein complexes. (DOC) [file pone.0138392.s001.doc]
